# Prevalence of *Leptospira interrogans* in wild rats (*Rattus norvegicus* and *Cricetomys gambianus*) in Zaria, Nigeria

**DOI:** 10.1016/j.heliyon.2021.e05950

**Published:** 2021-01-12

**Authors:** Collins Chimezie Udechukwu, Caleb Ayuba Kudi, Paul Ayuba Abdu, Elmina Abiba Abiayi, Ochuko Orakpoghenor

**Affiliations:** a Department of Veterinary Medicine, Ahmadu Bello University, Zaria, Nigeria; b Microbiology Division, Central Diagnostic Laboratory, National Veterinary Research Institute, Vom Plateau state, Nigeria; c Department of Veterinary Pathology, Ahmadu Bello University, Zaria, Nigeria

**Keywords:** Leptospirosis, Wild rats, Blood, Kidney, Urine

## Abstract

Leptospirosis is a neglected disease of zoonotic importance and rodents have a known role in epidemiology of *Leptospira* globally. Paucity of information on the prevalence of leptospirosis in wild rats used as games in Zaria, Nigeria informed the study. The study aimed to detect *Leptospira interrogans* in wild rats in Zaria, Nigeria. A total of 71 wild rats comprising 57 *Rattus norvegicus* and 14 *Cricetomys gambianus* were sampled over a period of 3 months (April–June 2019). Fisher exact test was used with confidence interval set at 0.05 to ascertain associations between positive cases and species. Blood was collected from 56 rats and harvested sera screened for *Leptospira interrogans* antibody using rat IgG competitive enzyme linked immunosorbent assay (c-ELISA). Following humane euthanasia of rats, 71 samples (62 kidney tissues and 9 urine samples) were collected in sterile labeled tubes and cultured using Ellinghausen Mc-cullough Johnson Harris (EMJH) enrichment and basal medium. Results indicated over all *Leptospira* spp antibody detection of 73.2 % (41/56) in *Rattus norvegicus* (60.7 %) and *Cricetomys gambianus* (12.5 %). No significant difference (P > 0.05) existed for the prevalence of *Leptospira interrogans* antibody in the species of wild rats. Over all occurrence of *Leptospira interrogans* were 74.2 % (46/62) in kidneys and 55.6 % (5/9) in urine samples. Based on species of rats, *Rattus norvegicus* recorded prevalence of 76.9 % (40/52) and 40.0 % (2/5) in kidney and urine samples respectively. Prevalence of 60.0 % (6/10) and 75.0 % (3/4) in kidney and urine samples respectively were recorded for *Cricetomys gambianus*. There was significant difference (P < 0.05) in the prevalence of *Leptospira interrogans* in kidney samples of both wild rats. These species of rats could be reservoirs of *Leptospira interrogans*. The result showed high prevalence of *Leptospira* spp in the wild rats and the possibility of domestic animals and humans contracting the disease. This study is the first documentation of evidence of pathogenic *Leptospira* species in wildlife used as games in Zaria, Nigeria.

## 1 Introduction

Leptospirosis is a disease of animals and humans caused by all pathogenic spirochete of the genus *Leptospira* and family *Leptospiraceae* (Bharti et al., 2003; Adler and de la Peña Moctezuma, 2010; Boey et al., 2019). The disease is the most widespread zoonosis and causes significant losses in the livestock industries (Ellis and Adler, 2015; Costa et al., 2015).

Over the years, wild rats have been increasingly recognised as the carrier host and environmental agent of spread of different pathogenic leptospires (Chin 2000; Cirone et al., 1978; Cox et al., 2005; Hamir et al., 2001). Increase in disease outbreak in domestic animals (particularly dogs) and change in serovars involved have been attributed to the endemicity of the disease in wild rats and increase in number of urban wild rats (Okewole and Ayoola 2009; Prescott et al., 2002).

In Nigeria, leptospirosis has been demonstrated serologically in wild rats (Diallo and Dennis 1982; Ajayi et al., 2017) and humans (Ezeh et al., 1991). Leptospirosis constitutes zoonotic risk, however, little information on its epidemiology and health risk in developing countries emanates from lack of awareness (Ajayi et al., 2017).

The diagnoses of leptospirosis have been achieved by serology, and isolation of *Leptospira* spp. from urine, blood and renal tissues (Adler et al., 1980; Adler and de la Peña Moctezuma, 2010; Toyokawa et al., 2011). Rats have been documented to be chronic asymptomatic carriers of different pathogenic serovars of *Leptospira* spp with capacity of causing disease in humans and other animals (Boey et al., 2019).

There is abundance of wild rats (*Rattus norvegicus* and *Cricetomys gambianus*) and paucity of information on detection and isolation of *Leptospira* spp in wild rats in Zaria, Nigeria and the possibility of people contracting the disease through improperly roasted game inspired us to investigate the prevalence of *Leptospira* spp in wild rats in Zaria, Nigeria.

## 2 Materials and methods

### 2.1 Study area

The study was conducted in Zaria, Nigeria positioned between latitude 11 07 N and 11 12 N and longtitude 07 41 E. Zaria is characterized by tropical climate, with a monthly mean temperature ranging from 13.8-36.70 °C and annual rainfall of 1092.8mm. Dry season farming is the second most prevalent agricultural activity in Zaria with vegetables being the common produce and cereals crops (Benedine and Ahmed, 2007).

### 2.2 Animals used for study

Wild rats (*Cricetomys gambianus* and *Rattus norvegicus*) found around farms, feed mills, grain markets and houses in Zaria, Nigeria were used for the study.

### 2.3 Sampling

Sampling of rats was based on purposive and convenience sampling technique i.e, rats were sampled at the particular time of capture. A total of 71 wild rats comprising 57 *Rattus norvegicus* and 14 *Cricetomys gambianus* were sampled over a period of 3 months (April–June 2019). The rats were captured from farms, feed mill, homes and grain market in Zaria, Nigeria using locally constructed traps made of iron baited with groundnuts and fish.

### 2.4 Samples collection

Blood sample was collected intra cardiac from 56 rats into labeled plain tubes and allowed to clot. Thereafter, serum was harvested and stored until used for *Leptospira interrogans* antibody detection.

The rats were then humanely euthanized, 62 kidney and 9 urine samples were collected and stored at a temperature of 4–8 °C until used for *Leptospira* spp culture.

### 2.5 Detection of *Leptospira interrogans* antibodies

The detection of *Leptospira interrogans* antibodies was done using commercially available rat IgG c-ELISA kit (Abbkine Scientific®, KTE100707, China) coated with specific *L*. *interrogans* antigen by following the manufacturer's protocol. The percent (%) inhibition for the samples was calculated. Samples with % Inhibition >40 % were considered positive and % Inhibition ≤40 % were considered negative according to the manufacturer's protocol.

### 2.6 *Leptospira* species culture and isolation

Ellinghausen McCullough Johnson Harris (EMJH) semi solid medium (Difco® USA) were used for the culture and isolation. The medium was prepared base on manufacturer's instructions for each sample (urine and kidney). The kidney and urine samples were dispensed into the medium as described by Oie et al. (1986). Tubes were incubated in the dark at 30 °C for 5–7 weeks following the method of Isenberg (1992) and Sakhaee et al. (2007). Thereafter, growths were examined using dark field microscope at X 400 magnification for presence of highly motile organism that measures about 6–25μm long and 0.1–0.2 μm in diameter is indicative of *Leptospira* organisms (Bharti et al., 2003).

### 2.7 Data analyses

Data obtained from both culture and ELISA was subjected to descriptive statistics and presented as percentages in tables. The prevalence of *Leptospira* spp was calculated for each sample and species using the formula outlined by Bennette et al. (1991). Fisher exact test for association between rat species was performed using statistical package for social scientist (SPSS version 20) and values of P ≤ 0.05 were considered significant.

### 2.8 Ethical clearance

Ethical approval for the use of animals in this study was granted by the Ahmadu Bello University Committee on Animal Use and Care (ABUCAUC).

## 3 Results

Out of the 56 serum samples tested, 41 (73.2 %) were positive for *Leptospira interrogans* antibodies, of which 34 (73.9 %) were from *Rattus norvegicus* and 7 (70.0 %) from *Cricetomys gambianus* (Table 1). There was no significant difference (P > 0.05) on the seroprevalence of *Leptospira interrogans* in the blood of the two rat species sampled.

**Table 1:** Prevalence of *Leptospira interrogans* in blood, kidney and urine samples of wild rats in Zaria, Nigeria.

Species of rat	Blood sample	Kidney sample	Urine sample
*R. norvegicus*	73.9 (46)	76.9 (52)∗	40.0 (5)∗
*C. gambianus*	70.0 (10)	60.0 (10)	75.0 (4)
Total	73.2 (56)	74.2 (62)	55.6 (9)

∗Showed significant difference at P < 0.05 using Fisher Exact Test.

From culture, of the 62 kidneys and 9 urine samples, 46 (74.2 %) and 5 (55.6 %) respectively were positive for *Leptospira* spp (Table 1). *Rattus norvegicus* recorded prevalence of 76.9 % (40/52) and 40.0 % (2/5) in kidney and urine samples respectively. Prevalence values of 60.0 % (6/10) and 75.0 % (3/4) in kidney and urine samples respectively were recorded for *Cricetomys gambianus* (Table 1). There was significant difference (P < 0.05) in the occurrence of *Leptospira interrogans* in kidney and urine tissue sample of the two rat species sampled (Table 1).

## 4 Discussion

In spite of the zoonotic risk of leptospirosis, little is known of the epidemiology and the health risks of the disease in developing countries, especially in Nigeria where game are regarded as a source of protein. This might be because of lack of awareness and the difficulty associated with the disease identification and diagnosis (Ajayi et al., 2017). Therefore the study is aimed to determine the prevalence of *Leptospira* spp in wild rats used as games in Zaria, Nigeria.

In this study, the detection of *Leptospira interrogans* in the sera, kidney and urine samples of wild rats in Zaria, Nigeria was carried out. The overall seroprevalence reported in this study was 73.2 %. This is higher than the 4.5 % by Diallo and Dennis (1982) in rats in Zaria, 8.44% by Ngbede (2012) in cattle and 15.83% by Adah and An (2013) in pigs in Kaduna State, Nigeria. The causes of the high seroprevalence (73. 2 %) observed in this studies are unknown but could be attributed to adverse climatic conditions which includes; long periods of rainfall and flooding, elevated temperatures all year round and high humidity may have favoured the seroprevalence of *Leptospira* spp in Zaria, Nigeria (Ajayi et al., 2017). These variations in seroprevalence could also be attributed to the type of test carried out as IgG c-ELISA detects IgG indicating chronic or latent infection was used in this study. However, in previous studies, detection targeted IgM antibodies to *Leptospira* which usually indicate current infection (Diallo and Dennis, 1982; Adah and An, 2013).

*Leptospira interrogans* was present in the kidney (74.2 %) and urine (55.6 %) of the sampled rats in this study. Ajayi et al. (2017) reported 78.1 % prevalence in cultured kidney samples of great crane, African giant, tree hyax, civet cat, monitor lizard, python bush buck and partridge; and 80.0 % prevalence in cultured kidney samples of African giant rat (AGR) on EMJH media in Abeokuta, Ogun State Nigeria. The lower prevalence observed in urine might be due to poor detection of *Leptospira* spp from urine samples of animals (Fearnley et al., 2008). This lower prevalence is as a result of intermittent shedding of *Leptospira* spp in urine as compared to kidney tissue sample which is the primary organ of colonization of *Leptospira* spp (Fang 2014). Also, obtaining urine was difficult as most rats spilled urine under stress, and this explains why some urine samples were not collected.

Specie related occurrence of leptospirosis in the kidney tissue samples of animals under investigation appears to be (P < 0.05) significant. The *Rattus norvegicus* showed more prevalence of *Leptospira* spp in the kidney tissue than in *Cricetomys gambianus*. This might have been because of the fact that *Leptospira* spp has more affinity on specific receptor (PRR) present in the kidney of *Rattus norvegicus* when compared to *Cricetomys gambianus* and hence lesser immune complex activation to clear *Leptospira* spp in *Rattus norvegicus* than in *Cricetomys gambianus* (Akira et al., 2006). The result obtained here contradicts the work of Ajayi et al. (2017) who observed prevalence of 80% in *Cricetomys gambianus.*

The high prevalence observed in this study may be as a result of high abundance of rat in unhygienic premises around houses and farms where most of the rats dwell, thereby contributing to the risk of infection. Rats are asymptomatic carrier of *Leptospira* spp and are the most important known source of the infection. Infected rats become carriers for life and continue to shed the agent (Leonard et al., 1992; Bharti et al., 2003).

## 5 Conclusion

The result of this study has shown that *Leptospira interrogans* is prevalent among wild rats population in Zaria, Nigeria. In kidney tissue of *R. norvegicus*, the prevalence of *Leptospira* spp was higher compared to that of *C. gambianus*. The high prevalence of leptospirosis in this study showed the endemicity of the disease in the wild rats in Zaira, Nigeria, and might be a potential source of infection to domestic animals and humans who used wild rats as games.

### 5.1 Limitation of study

Urine collection in wild rats was difficult as most rats spills urine under stress hence making urine sample collection difficult. Rat capture and acclimatisation was difficult as most rat died in captivity.

## Declarations

### Author contribution statement

C. A Kudi, P.A Abdu: Conceived and designed the experiments.

C. C Udechukwu: Performed the experiments; Analyzed and interpreted the data; Wrote the paper.

O. Orakpoghenor, E. Abiba: Contributed reagents, materials, analysis tools or data.

### Funding statement

This work was supported by 10.13039/501100014417National Veterinary Research Institute (NVRI), Nigeria.

### Data availability statement

Data will be made available on request.

### Declaration of interests statement

The authors declare no conflict of interest.

### Additional information

No additional information is available for this paper.
